# Supplementary figures and images for: Pleiotropic Effects of ebony and tan on Pigmentation and Cuticular Hydrocarbon Composition in Drosophila melanogaster
Source: Front Physiol. 2019 May 1;10:518. doi: 10.3389/fphys.2019.00518 (PMC6504824; doi:10.3389/fphys.2019.00518)

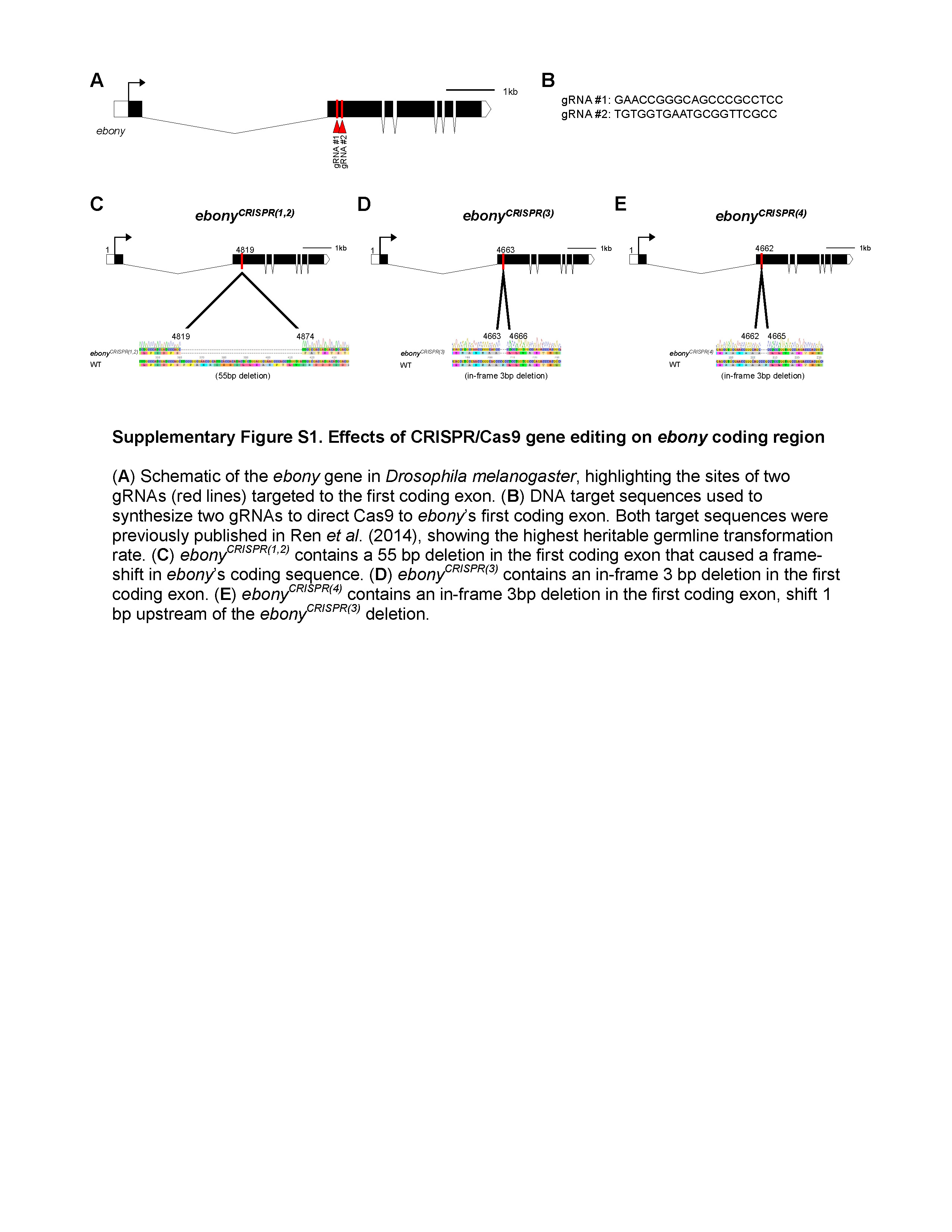

Supplement: Supplementary file 2 [file Image_1.JPEG]

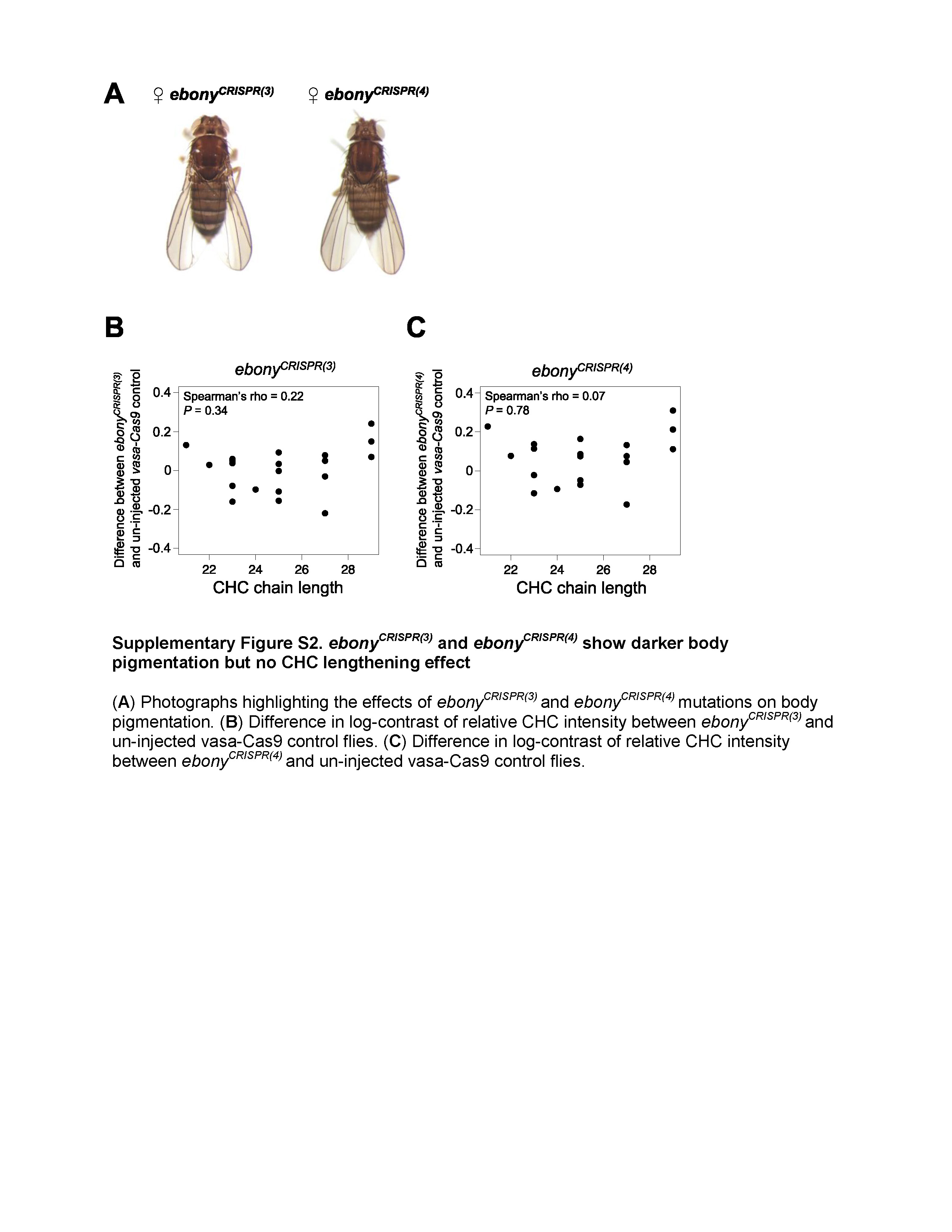

Supplement: Supplementary file 3 [file Image_2.JPEG]

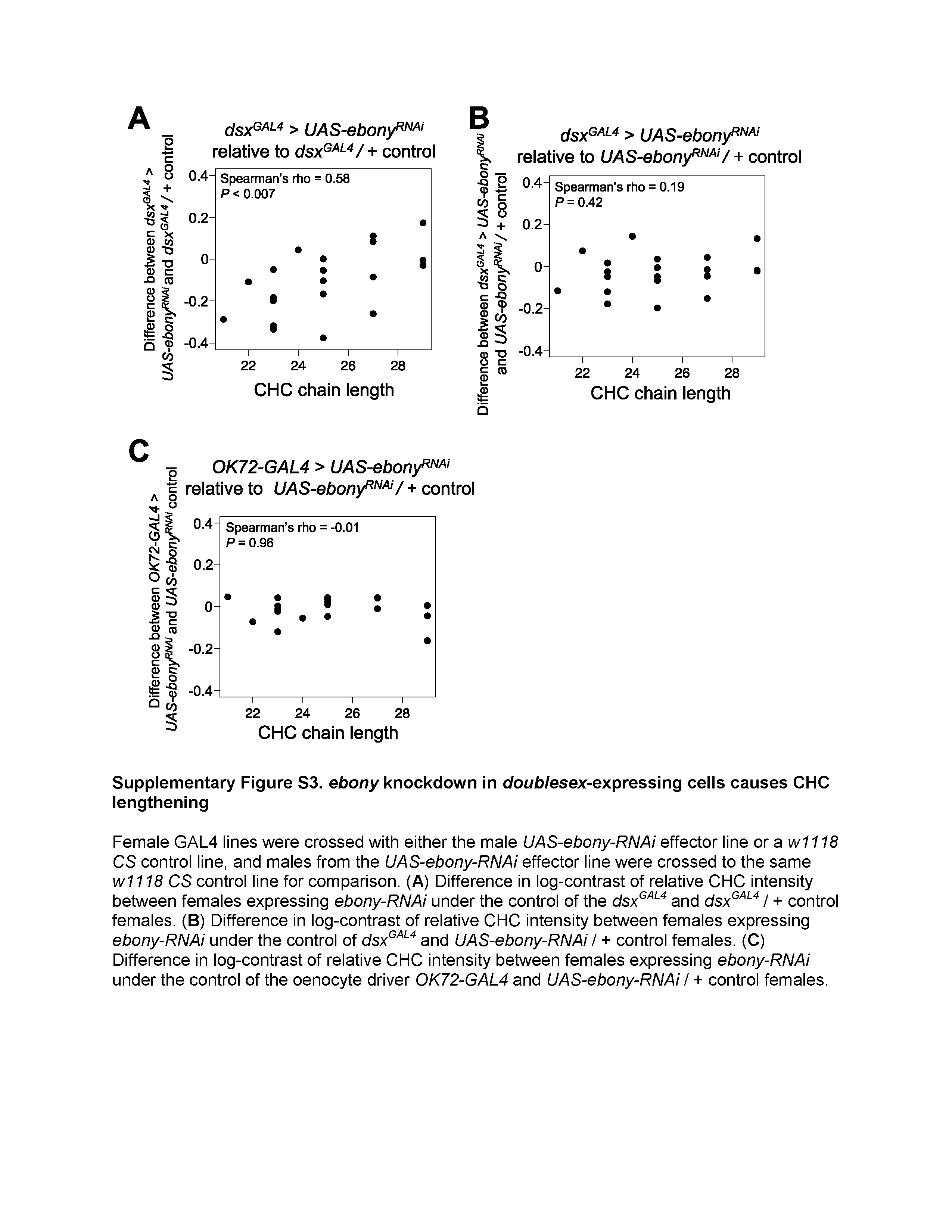

Supplement: Supplementary file 4 [file Image_3.JPEG]

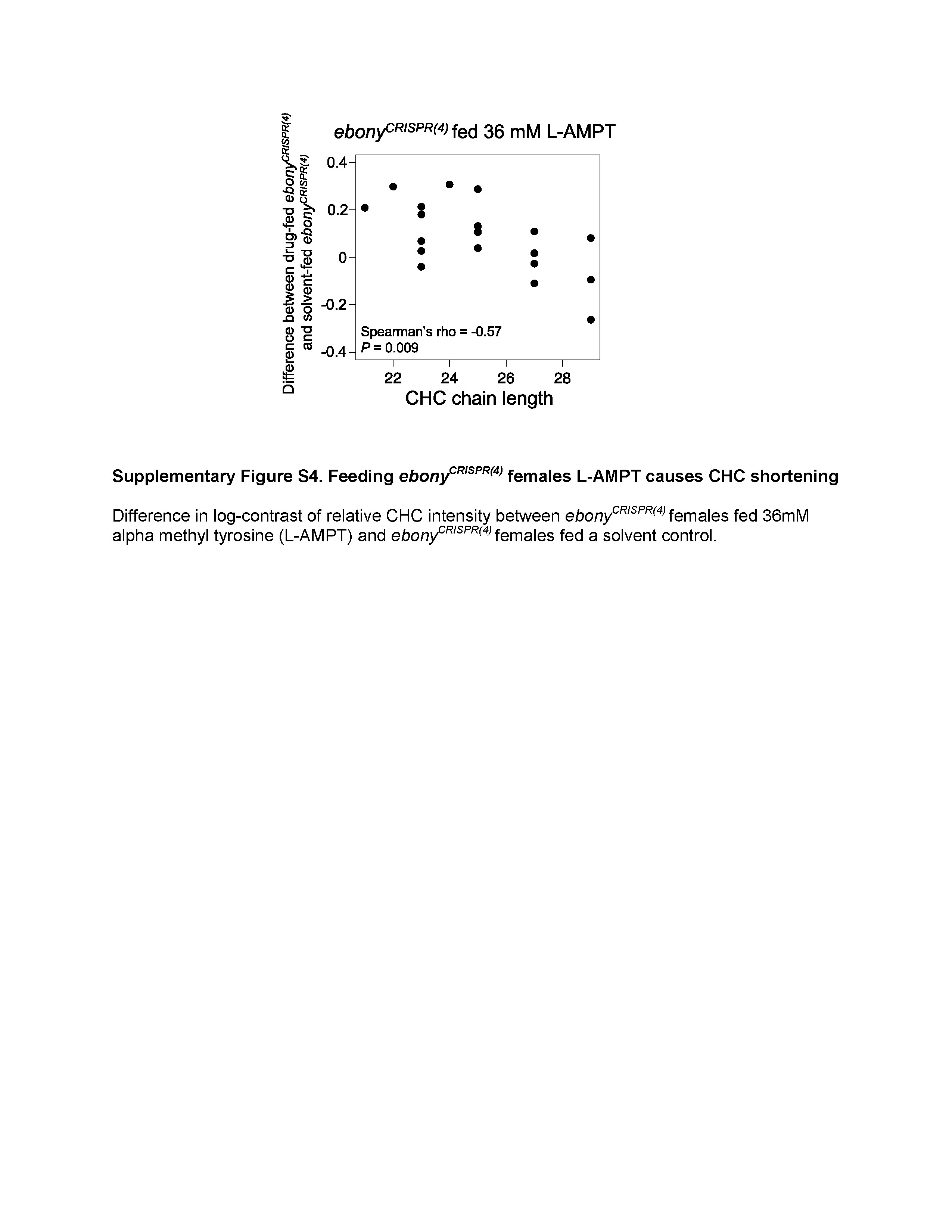

Supplement: Supplementary file 5 [file Image_4.JPEG]

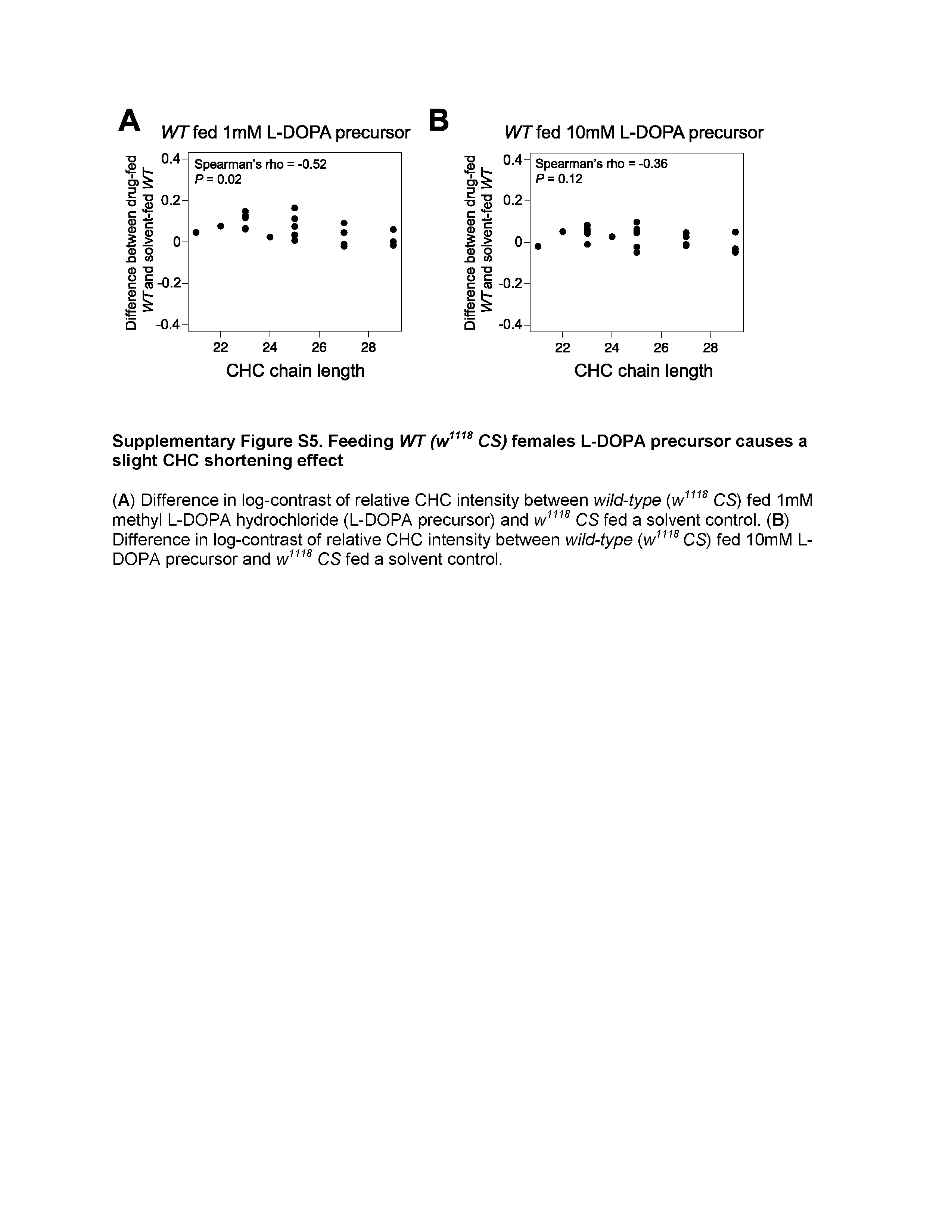

Supplement: Supplementary file 6 [file Image_5.JPEG]

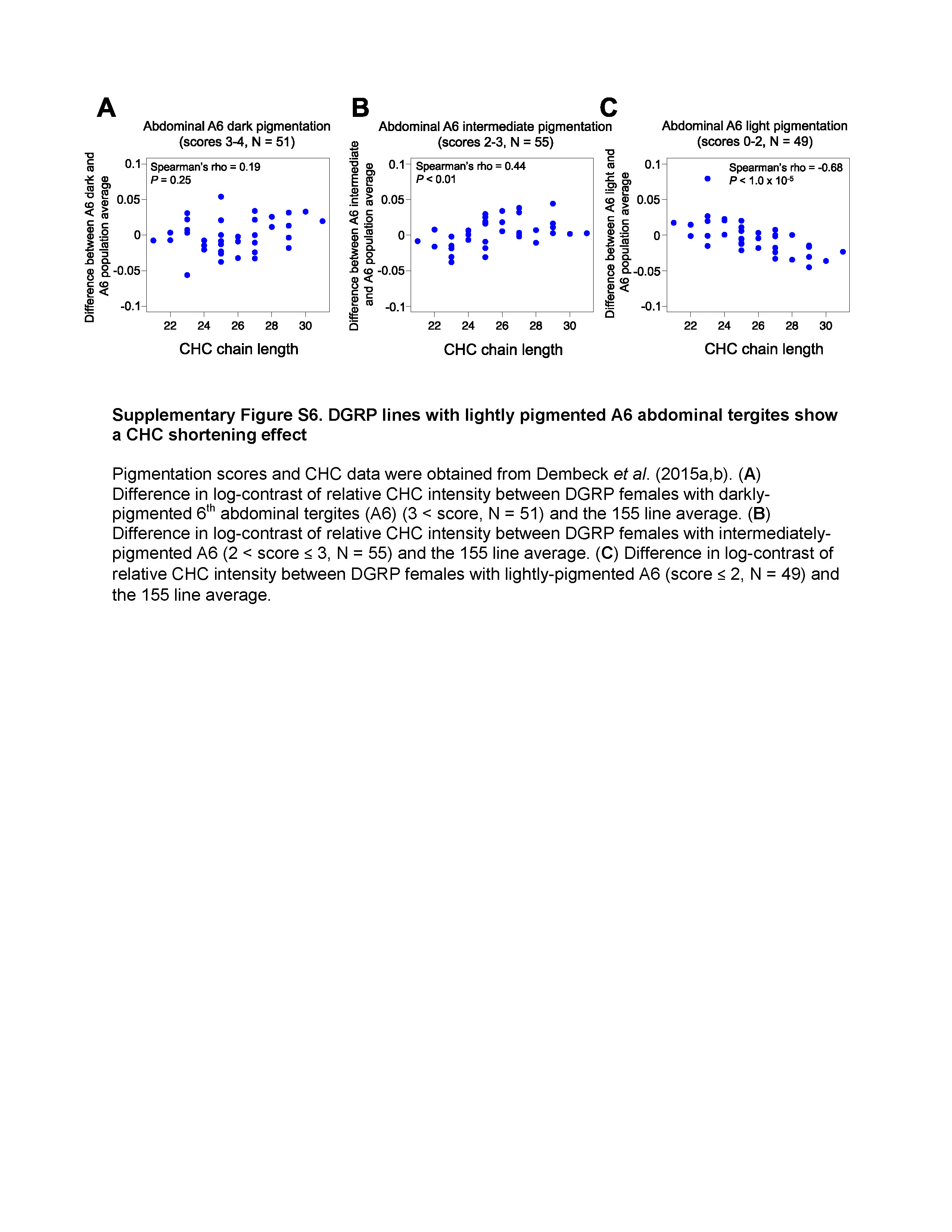

Supplement: Supplementary file 7 [file Image_6.JPEG]
